# Practical consequences of model misfit when using rating scales to assess the severity of attention problems in children

**DOI:** 10.1002/mpr.1795

**Published:** 2019-07-01

**Authors:** Daniela R. Crișan, Jorge N. Tendeiro, Rob B.K. Wanders, Don van Ravenzwaaij, Rob R. Meijer, Catharina A. Hartman

**Affiliations:** ^1^ Department Psychometrics and Statistics, Faculty of Behavioral and Social Sciences University of Groningen Groningen The Netherlands; ^2^ Department of Psychiatry University Medical Center Groningen Groningen The Netherlands

**Keywords:** attention problems score estimates, CBCL, consequences of model violations, item response theory, TRAILS

## Abstract

**Objectives:**

In this study, we examined the consequences of ignoring violations of assumptions underlying the use of sum scores in assessing attention problems (AP) and if psychometrically more refined models improve predictions of relevant outcomes in adulthood.

**Methods:**

Tracking Adolescents' Individual Lives data were used. AP symptom properties were examined using the AP scale of the Child Behavior Checklist at age 11. Consequences of model violations were evaluated in relation to psychopathology, educational attainment, financial status, and ability to form relationships in adulthood.

**Results:**

Results showed that symptoms differed with respect to information and difficulty. Moreover, evidence of multidimensionality was found, with two groups of items measuring sluggish cognitive tempo and attention deficit hyperactivity disorder symptoms. Item response theory analyses indicated that a bifactor model fitted these data better than other competing models. In terms of accuracy of predicting functional outcomes, sum scores were robust against violations of assumptions in some situations. Nevertheless, AP scores derived from the bifactor model showed some superiority over sum scores.

**Conclusion:**

These findings show that more accurate predictions of later‐life difficulties can be made if one uses a more suitable psychometric model to assess AP severity in children. This has important implications for research and clinical practice.

## 1 INTRODUCTION

The Child Behavior Checklist (CBCL/6–18; Achenbach, 1991a; Achenbach, Dumenci, & Rescorla, 2003) is an inventory often used in practice to assess children on behavioral and emotional problems and competencies, including attention problems (AP). Due to the broad range of child behavior and psychopathology assessed, the CBCL/6–18 is a popular instrument in research (e.g., Chen et al., 2016) and clinical context (e.g., Raiker et al., 2017).

The Attention Problems Syndrome Scale is one of CBCL's empirically based scales and, it is used to assess the extent to which children show symptoms of AP. Graetz, Sawyer, Hazell, Arney, and Baghurst (2001) showed that scores on the AP scale are strongly associated with diagnoses of attention deficit hyperactivity disorder (ADHD)–inattentive subtype. This indicates that the AP scale significantly discriminates between ADHD inattentive and hyperactive/impulsive diagnoses. Other studies also demonstrated the sensitivity, specificity, predictive power, and clinical utility of the AP scale for an ADHD diagnosis (e.g., Raiker et al., 2017), as well as its convergence with other established ADHD rating scales (e.g., Kasius, Ferdinand, van den Berg, & Verhulst, 1997).

The sum scores on the CBCL's AP scale are used for scoring individuals with respect to symptom severity and, based on predefined cutoff scores, for a provisional categorization of “probable ADHD.” As we will discuss below, an alternative is to use scores based on more refined models, such as item response theory (IRT) models (e.g., Embretson & Reise, 2000). These scores provide more detailed information about severity of AP symptoms and also may improve prediction of later‐life functional outcomes. In IRT, scores are interpreted by comparing their distance from items (item‐referenced meaning) rather than by comparing their positions in a normally distributed reference group (norm‐referenced meaning; Embretson & Reise, 2000, p. 25). Norm‐referenced scores do not inform the clinician about which symptoms are a person more likely to develop, whereas item‐referenced scores do. This is possible because individual IRT‐derived AP scores and symptom properties are placed on the same dimension. Individual severity scores can thus be directly linked to the probabilities of developing specific symptoms.

The main aim of this study was to determine the potential advantages of using more refined scores for the assessment of AP severity in relation to functional outcomes. We also wanted to assess how problematic the common use of sum scores was in situations where the measurement model did not fit the data well.

### 1.1 Using sum scores to assess AP severity

AP scales are commonly scored using the principles of classical test theory (CTT; Lord & Novick, 1968). In CTT, the observed score, usually obtained by summing individuals' responses to items, is used as an estimate of the individual's true score. The use of sum scores as proxies for the true scores assumes that variation on each item is caused by a single general factor (unidimensionality/homogeneity assumption) and that measurement error is equal across all scores in a population (i.e., all individuals are measured with the same precision).

Achenbach (1991a) derived the CBCL syndrome scales by imposing orthogonality of the syndromes and by forcing the items with large cross‐loadings to load on only one domain. This approach ignores the fact that domains of child psychopathology are highly correlated (e.g., Angold, Costello, & Erkanli, 1999) and that some items measure more than one dimension (multidimensionality). Empirical studies showed that imposing such restrictions on the data leads to poor model fit and large cross‐loadings, indicating model misspecification (e.g., Hartman et al., 1999; Van den Oord, 1993) and difficulties in interpreting CBCL sum scores as unidimensional indicators of psychopathology (Kamphaus & Frick, 1996). Regarding ADHD, for example, a two‐factor structure (i.e., inattention and hyperactivity/impulsivity) received the widest support before the year 2000 (Willcutt et al., 2012). Since 2000, the bifactor model of ADHD has received vast support, with ADHD as a general factor and specific factors for inattention and hyperactivity/impulsivity (e.g., Caci, Morin, & Tran, 2016). More recently, there has been considerable interest in whether sluggish cognitive tempo (SCT), a construct comprising symptoms such as daydreaming, confusion, and apathy (e.g., Becker, Burns, Schmitt, Epstein, & Tamm, 2017; Hartman, Willcutt, Rhee, & Pennington, 2004) is a dimension of ADHD or a separate psychopathology. Lee, Burns, Beauchaine, and Becker (2016) and Garner et al. (2017) found support, through bifactor modeling, for SCT as a distinct construct, although strongly and positively correlated with inattention. Additionally, studies on the Youth Self‐Report form of the CBCL (Lambert et al., 2003; Lambert, Essau, Schmitt, & Samms‐Vaughan, 2007) showed that AP symptoms differ in their level of measurement precision.

Despite these findings of multidimensionality and differences in measurement precision across items, users of the CBCL's AP scale often do not take this into account: A single unweighted sum score is still commonly used to summarize responses. However, the sum score on a scale that violates the assumptions of unidimensionality and equal measurement precision may not accurately reflect a person's true AP severity.

### 1.2 IRT as a psychometric tool for assessing AP

Modern approaches based on IRT have been used less often than confirmatory factor analysis to understand and improve the assessment of AP. IRT is a modern paradigm for the construction, analysis, and scoring of tests and questionnaires. This robust approach is preferred over CTT due to its “more theoretically justifiable measurement principles and the greater potential to solve practical measurement problems” (Embretson & Reise, 2000, p. 3). One of the advantages of IRT over confirmatory factor analysis is that most IRT models consider the complete response patterns when estimating individual scores. One implication, which also applies to the assessment of AP, is that individuals with the same sum score can have different IRT‐derived severity levels. Another advantage of IRT is that the score's standard error of measurement is conditional on the person's severity level as estimated by the model. In fact, one of the measurement principles of IRT is that some individuals can be measured with higher precision than others by a set of symptoms. In short, IRT provides more detailed information at any value of AP than sum scores do.

Applications of IRT to AP assessment have mostly focused on scale construction/revision and analysis, but little has been done with respect to using IRT models to improve the *scoring* of individuals. One exception is the work of Dumenci and Achenbach (2008) who found a strong nonlinear association between IRT‐ and CTT‐derived scores, implying that sum scores are biased towards the ends of the trait continuum for Likert‐type data. This has major implications in clinical practice, where important decisions are made based on very high or very low scores. Typically, IRT was used for purposes such as differential item functioning analysis (e.g., Flora, Curran, Hussong, & Edwards, 2008; Lambert et al., 2007; Stevanovic et al., 2017), test score linking (e.g., Kaat et al., 2018), item selection (Lambert et al., 2003), or examining item properties over time (e.g., Petersen, Bates, Dodge, Lansford, & Pettit, 2016). These empirical studies showed that symptoms differ with respect to the information (related to measurement precision) they provide across the severity continuum and with respect to their level of difficulty (i.e., some symptoms are endorsed more often than others).

### 1.3 Present study

In the present study, we focus on the potential advantages of using IRT models for *scoring* individuals on the AP severity continuum. We extend the study of Dumenci and Achenbach (2008) by looking not only at the association between different types of score estimates but also at their accuracy of predicting functional outcomes measured more than 10 year later. As Dumenci and Achenbach (2008, p. 61) argued, using scoring methods that are not suited to fit Likert‐type data is detrimental for inferences from longitudinal studies. As such, we first investigated the psychometric characteristics of the CBCL's AP scale at age 11, choosing the model that described the data best. Second, we investigated the practical implications, in terms of functional consequences, of using a more refined psychometric model to assess the severity of AP symptoms, by comparing sum scores to scores derived from the best fitting IRT model. We investigated the possible benefit of a psychometrically improved scale using functional outcomes at age 22 as a criterion, long after the first measurement of AP (at age 11). Because IRT models imply a more complex scoring strategy, it is relevant to assess whether the gains outweigh the added model complexity. An important contribution of this study is that the functional outcomes that we tried to predict were measured more than 10 years after the predictor was measured. Given this large time gap between measurements, any gain in predictive accuracy is extremely valuable and renders the use of psychometrically superior models worthwhile.

Given the mixed findings in the literature with respect to the factor structure of the CBCL problems domains, we refrained from advancing specific hypotheses regarding the dimensionality of the AP scale, and we favored an exploratory approach. Concerning the predictive accuracy of the different scoring methods, we hypothesized that IRT‐derived AP scores would have higher accuracy compared with CTT sum scores. Evidence collected to study our hypothesis includes several categories of difficulties associated with childhood AP.

## 2 METHODS

### 2.1 Sample

We analyzed data from the TRacking Adolescents' Individual Lives Survey (TRAILS; Oldehinkel et al., 2015), a large longitudinal study conducted in the Netherlands starting in 2001, with five assessment waves (T1 through T5) completed thus far (for a more detailed description of the TRAILS design and of the first four waves, consult Oldehinkel et al., 2015). TRAILS consists of two prospective cohorts: a population‐based cohort (2,230 participants at T1) and a clinical cohort, starting roughly 2 years later and consisting of 543 children at T1 who were referred to a psychiatric specialist before the age of 11. Mean age at T1 was 11 years in both cohorts. The fifth measurement wave (T5) was completed between 2012 and 2013 (population cohort) and between 2015 and 2017 (clinical cohort) and had a retention rate of 80% of the baseline sample in the population cohort and 74% in the clinical cohort. Mean age at T5 was 22 years in both cohorts.

We used data from the first measurement wave (T1) and from the fifth measurement wave (T5). Data at T2 were used to compute the test–retest reliability of the CBCL AP scale. Respondents with missing values on more than half of the items were removed, which resulted in a dataset of 1,642 respondents in total. The percentage of missing values per variable was smaller than 5% and 7% at T1 and T5, respectively. The mice package (Van Buuren & Groothuis‐Oudshoorn, 2011) in R (R Development Core Team, 2017) was used to impute the missing values.

### 2.2 Measures—CBCL/6–18 AP scale

TRAILS uses the CBCL/6–18 battery. For this study, we used CBCL's empirically based Attention Problems Syndrome Scale, consisting of 10 symptoms rated on a 3‐point Likert scale ranging from 0 to 2 (0 = *Not true*; 1 = *Somewhat or sometimes true*; 2 = *Very true or often true*). These symptoms refer to day‐to‐day behavior, like engaging in school work or play activities. Parents rate the behavior of their child for each symptom. The individual scores are then summed to obtain a continuous measure of AP severity. In the original sample (i.e., before removing cases due to missing values), the test–retest correlations (.66 and .70 in the population and clinical cohort) and Cronbach's alpha (.81 and .76 across cohorts) showed adequate score reliability.

### 2.3 Measures—Outcomes

#### 2.3.1 Psychopathology

The self‐reported Attention Problems (15 symptoms), Internalizing Problems (39 symptoms), and Externalizing Problems (35 symptoms) from the Adult Self‐Report version of the CBCL were also included in the TRAILS survey and were used as long‐term outcomes at T5. Research showed that individuals who suffer from attention disorders (ADHD in particular) tend to experience these kinds of difficulties in adulthood (e.g., Molina & Pelham, 2014). In clinical practice, a total score for each outcome is obtained by summing the individual symptom scores, after which categories of symptom severity are obtained based on gender‐specific cutoff values (Achenbach & Rescorla, 2001; see Table S1).

#### 2.3.2 Other outcome measures

We also considered the participants' ability to function in several life areas as young adults, with the following specific areas measured with the TRAILS survey at T5: (a) education achievement—a single question asking participants to indicate their latest obtained diploma by choosing one of the 15 available options representative for different levels of education in the Netherlands. Subsequently, these were categorized into four categories representing lower or vocational education (e.g., Dutch VMBO “voorbereidend middelbaar beroepsonderwijs” and KMBO “kort middelbaar beroepsonderwijs”), middle (Dutch MBO “middelbaar beroepsonderwijs”), middle to higher (Dutch HAVO “hoger algemeen voortgezet onderwijs” and VWO “voorbereidend wetenschappelijk onderwijs”), and higher education (e.g., Dutch HBO “hoger beroepsonderwijs”); (b) work/financial situation/independence from parents was operationalized by the following variables: living outside parental home (yes/no), whether the person ever had a paid job (yes/no), monthly income (low: €300–€600; low to middle: €601–€900; middle: €901–€1,200; middle to high: €1,201–€1,800; High: >€1,801), and whether the person benefits from a form of Dutch social security aid (Dutch Bijstand or Wajong); (c) romantic relationships status was operationalized by whether the person was ever involved in a romantic relationship (yes/no).

### 2.4 Outline of the analyses

The following analyses were conducted. First, on the AP data (for both cohorts separately) at T1, we investigated whether there were violations of the assumptions underlying the use of sum scores. Second, we investigated whether such violations had practical implications on outcomes at T5. The presence of violations and poorly functioning symptoms was investigated through a combination of methods from CTT (e.g., principal component analysis [PCA], parallel analysis, and corrected item‐total correlations) and IRT (e.g., the graded response model, GRM; Samejima, 1969).

We estimated three IRT models that, from a psychometric perspective, may describe the data better: the *unidimensional* GRM, the *multidimensional* GRM, and the full‐information bifactor model. We used the R package mirt (Chalmers, 2012) to fit these models. Several exact and approximate goodness of fit measures were inspected in order to obtain a more informative picture of model fit (Maydeu‐Olivares, 2014): *M2* ^*^ limited information statistic, root mean square error of approximation, standardized root mean square residual, comparative fit index and Tucker–Lewis index, Akaike information criterion, and Bayesian information criterion (see Supporting Information for a description of the models and fit indices).

The practical implications of the existing violations were investigated by comparing the predictive accuracy of AP severity scores obtained from the optimal IRT model to the traditional CBCL sum scores and to unidimensional IRT scores. We constructed receiver operating characteristic plots and computed areas under the curve (AUCs) to compare how well sum scores and IRT‐derived scores at T1 can predict outcomes at T5. The goal was to compare the predictive accuracy of sum scores with IRT‐based person scores to classify persons, according to the previously mentioned various criteria at T5. We decided to analyze these predictions only on the clinical cohort, because these individuals represent a high‐risk group for experiencing all sorts of difficulties in functioning compared to the normal population cohort.

## 3 RESULTS

### 3.1 Sample descriptives

Descriptive statistics for the variables included in this study are presented separately by cohort and gender. At T1, the average sum score on the 10 CBCL AP symptoms was 3.5 (*SD* = 3.0) for girls in the population cohort, 7.5 (*SD* = 4.3) for girls in the clinical cohort, 4.6 (*SD* = 3.5) for boys in the population cohort, and 8.8 (*SD* = 3.6) for boys in the clinical cohort. Descriptive statistics of the outcome variables at T5 are presented in Table 1, for the clinical cohort.

**Table 1:** Number of cases and frequency of each outcome variable at T5 in the clinical cohort, separately by gender

Outcome	Gender			
	Females		Males	
	*n*	%	*n*	%
Attention clinical	11	10.6	6	3.2
Internalizing clinical	27	26.0	29	15.6
Externalizing clinical	3	2.9	11	5.9
Education lo*w*/*v*ocational/middle	80	76.9	118	63.4
Living with parents	39	37.5	109	58.6
No paid job	13	12.5	28	15.1
Low/low–middle income	75	72.1	119	64.0
Social benefits	25	24.0	46	24.7
Single	19	18.3	66	35.5
Total^a^	104	35.9	186	64.1

a The row named Total shows the total numbers and percentages of females and males across cohorts.

### 3.2 Model violations and psychometric evidence against interpreting sum scores as unidimensional indicators of AP severity

Table 2 shows descriptive statistics for individual symptoms and for the entire scale, across cohorts, at T1. Reliability estimates (test–retest correlations and Cronbach's alpha) were acceptable.

**Table 2:** CBCL's Attention Problems Syndrome Scale: Symptom and scale descriptive statistics at T1

Description^a^	Population cohort		Clinical cohort	
	(*N* = 1,352, *α* = .79)		(*N* = 290, *α* = .77)	
	*M*_item_	*r*_item rest_	*M*_item_	*r*_item rest_
Acts too young for his/her age (CBCL1)	0.33	.36	0.82	.36
Fails to finish things he/she starts (CBCL4)	0.69	.49	1.08	.48
Cannot concentrate, cannot pay attention for long (CBCL8)	0.55	.68	1.19	.63
Cannot sit still, restless, or hyperactive (CBCL10)	0.46	.49	1.09	.46
Confused or seems to be in a fog (CBCL13)	0.08	.32	0.28	.39
Daydreams or gets lost in his/her thoughts (CBCL17)	0.53	.33	0.84	.26
Impulsive or acts without thinking (CBCL41)	0.52	.57	1.04	.51
Poor school work (CBCL61)	0.19	.43	0.41	.32
Inattentive or easily distracted (CBCL78)	0.55	.71	1.23	.65
Stares blankly (CBCL80)	0.10	.24	0.33	.28
Mean (*SD*)	3.98 (3.24)		8.31 (3.92)	

Abbreviations: *α*, Cronbach's alpha; CBCL, Child Behavior Checklist; *M*_item_, item mean; *N*, sample size; *r*_item rest_, corrected item‐total correlation.

a Description of each item with original numbering in parentheses.

#### 3.2.1 PCA and parallel analysis

Both PCA with oblimin rotation and parallel analysis suggested two main components for both cohorts (see Table 3 for the distribution of symptoms across components).

**Table 3:** Principal component analysis loadings across cohorts

Symptom	Population cohort		Clinical cohort	
	PC1	PC2	PC1	PC2
CBCL1	.390	.288	.171	.597
CBCL4	.709		.771	
CBCL8	.932		.895	
CBCL10	.843	−.199	.764	
CBCL13	.299	.597	.231	.646
CBCL17		.816		.781
CBCL41	.707	.143	.709	
CBCL61	.579	.229	.528	
CBCL78	.852	.113	.832	
CBCL80		.903		.829

*Note*. Grey cells denote component correspondence.

Abbreviations: CBCL, Child Behavior Checklist; PC1, first component; PC2, second component.

The symptoms in the first component tap into ADHD symptoms of inattention and hyperactivity/impulsivity, and the symptoms in the second component tap into behavior that can be qualified as SCT. Interestingly, CBCL1 (“Acts too young for his/her age”) loaded inconsistently on the components and had very low communalities across cohorts: 31% and 46%, respectively. The correlation between the two components was rather small in both cohorts (about *r* = .3).

#### 3.2.2 IRT analyses

**Figure 1:**
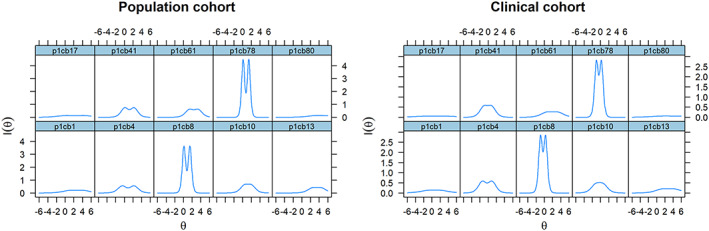
Information functions for the Child Behavior Checklist symptoms obtained with the unidimensional graded response model (exploratory), in the population cohort (left panel) and in the clinical cohort (right panel). θ denotes the latent trait continuum (i.e., severity of attention problems)

The previous results were corroborated by the results from IRT analysis (unidimensional GRM). In particular, these IRT analyses showed that not all symptoms are equally informative and that they do not imply the same probability of endorsement (see Table 4 and Figure 1).

**Table 4:** Discrimination (a) and threshold (b1, b2) parameters estimated with the unidimensional graded response model (exploratory), across cohorts

Symptom	Population cohort			Clinical cohort		
	a	b1	b2	a	b1	b2
CBCL1	0.876	1.106	4.199	0.709	−0.712	2.010
CBCL4	1.476	−0.450	2.268	1.519	−1.380	0.944
CBCL8	3.809	0.115	1.489	3.356	−0.912	0.307
CBCL10	1.574	0.473	1.994	1.346	−1.064	0.588
CBCL13	1.261	2.578	4.427	0.883	1.431	4.277
CBCL17	0.717	0.137	4.443	0.453	−1.434	3.310
CBCL41	1.711	0.117	2.276	1.485	−0.968	0.809
CBCL61	1.556	1.410	3.373	1.003	0.714	3.237
CBCL78	4.234	0.110	1.423	3.328	0.985	0.211
CBCL80	0.733	3.453	7.538	0.492	1.740	7.740

Abbreviations: a, discrimination parameter; b1, first threshold parameter; b2, second threshold parameter; CBCL, Child Behavior Checklist.

Figure 1 shows the information functions for the 10 CBCL symptoms. The plot indicates the measurement precision of the AP scale, across symptoms and severity continuum. The steepness of these curves is related to the values of the item discrimination parameters in Table 4: Steeper curves correspond to larger discrimination values and higher measurement precision, whereas flatter curves correspond to smaller discrimination values and higher measurement error. The threshold parameters, which determine the items location along the AP dimension, varied greatly. The most often endorsed symptoms according to the model are CBCL4 (population cohort) and CBCL17 (clinical cohort). The least endorsed symptom according to the model is CBCL80 in both cohorts. As an illustration of how IRT location parameters relate to AP severity, a symptom severity level of 1.74 standard deviations above the mean is necessary for an individual in the clinical cohort to answer at least 1 to CBCL80, with 4.1% of the individuals being expected to endorse this symptom.

Taken together, these results show that the CBCL symptoms differ with respect to the level of information they provide to measuring AP severity. Moreover, based on the results of the PCA, the symptoms violated the assumption of unidimensionality/homogeneity, and one symptom (put symptom here) was performing very poorly. The finding of multidimensionality is not surprising, because items CBCL13, CBCL17, and CBCL80 are part of a set of symptoms that is often used to assess SCT (Becker et al., 2017).

**Figure 2:**
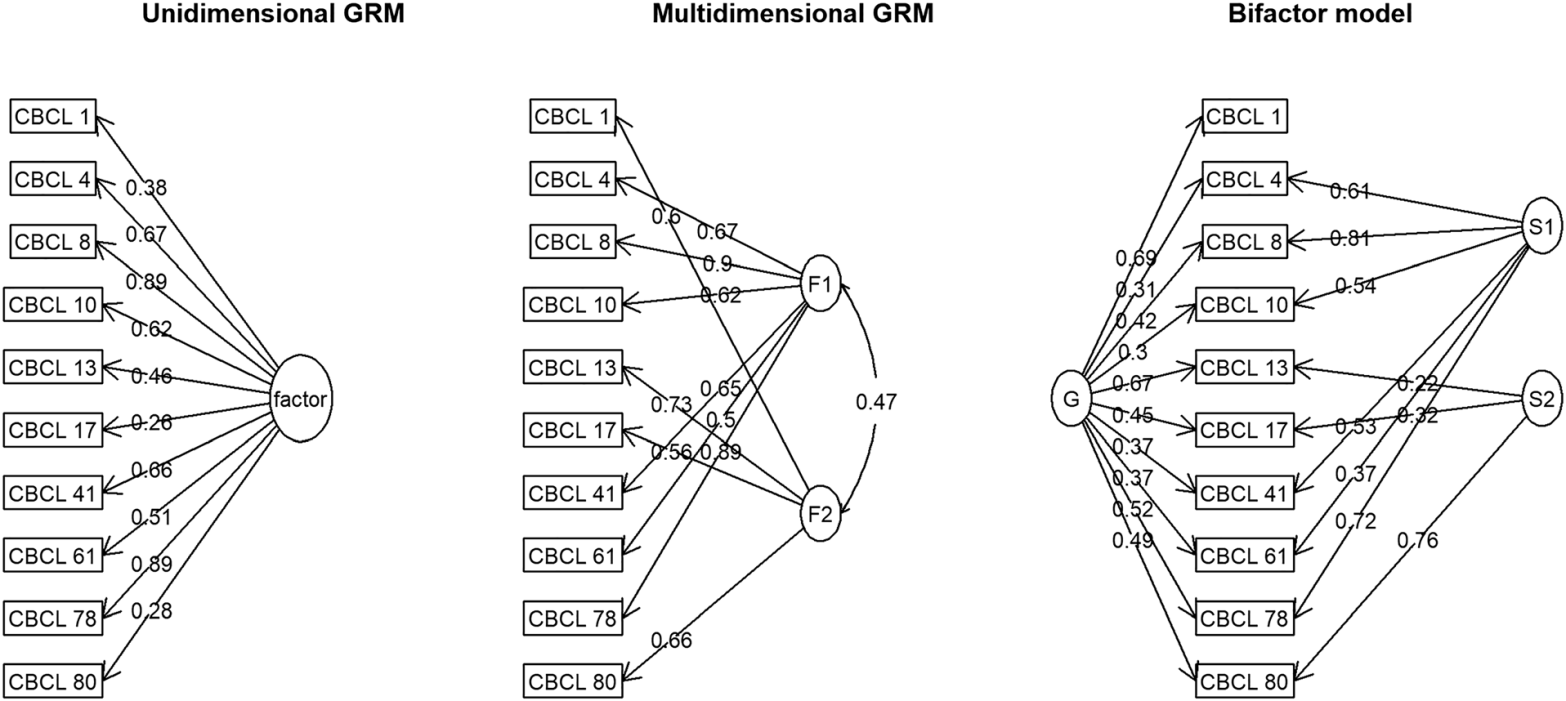
Confirmatory item response theory models fitted to the Attention Problems Syndrome Scale at T1, in the clinical cohort. CBCL, Child Behavior Checklist; G, general factor; GRM, graded response model; S1/S2, specific/group factors; values represent standardized factor loadings

Figure 2 shows the graphical displays of the three IRT models fitted to the data in the clinical cohort at T1. Because CBCL1 consistently showed low discrimination in the exploratory analyses, we constrained it to load only on the general factor (*G*) of the bifactor model, with zero loadings on the specific/group (*S1*/*S2*) factors. Table 5 shows the fit statistics corresponding to these models. When comparing the rows, we conclude that the bifactor model fits the data best, as indicated by decreasing values of *M2* ^*^, root mean square error of approximation, and standardized root mean square residual and increasing values of comparative fit index and Tucker–Lewis index.

**Table 5:** Model fit statistics for the Attention Problems Syndrome Scale

Model	*M2* ^*^; *df*; *p*	AIC	BIC	CFI	TLI	RMSEA	95% CI RMSEA	SRMSR
Unidimensional GRM	97.1; 25; <.001	5037.77	5147.9	0.87	0.82	0.10	(0.08; 0.12)	0.09
Two‐dimensional GRM	48.3; 24; .002	4970.2	5084.0	0.96	0.94	0.06	(0.03; 0.08)	0.06
Bifactor model^a^	26.7; 16; .045	4973.6	5116.8	0.98	0.96	0.05	(0.01; 0.08)	0.05

*Note*. Most favorable model fit highlighted.

Abbreviations: AIC, Akaike information criterion; BIC, Bayesian information criterion; CFI, comparative fit index; *df*, degrees of freedom associated to *M2* ^*^; GRM, graded response model; *M2* ^*^, goodness of fit statistic; *p*, significance level associated with *M2* ^*^; RMSEA, root mean square error of approximation; SRMSR, standardized root mean square residual; TLI, Tucker–Lewis index; 95% CI RMSEA, 95% confidence interval for RMSEA.

a Full information bifactor model with CBCL 4, 8, 10, 41, 61, and 78 on the first specific factor, CBCL 13, 17, and 80 on the second factor, and CBCL 1 on the general factor only.

In sum, we conclude the following: (a) There is evidence of multidimensionality in the data, indicating that the 10 symptoms measure a complex and heterogeneous construct. A bifactor model fits the data better than a unidimensional model or a two‐dimensional model with correlated factors. This suggests that although both dimensions are indicative of the same general or target construct, they are also distinct from one another; (b) symptoms differ with respect to their level of measurement precision; (c) there is one symptom, CBCL1, that functions poorly within the scale.

On the basis of these analyses, it is clear that the structure of the data of the CBCL's AP scale may be better represented by estimates from a more complex psychometric model than by a simple sum score. The next question then is whether using IRT‐based scoring has any added practical advantages over sum scores.

### 3.3 Practical consequences of ignoring model violations on the predictive accuracy of long‐term outcomes

In order to evaluate our hypothesis, we compared the predictive accuracy of AP severity estimates using sum scores, person estimates derived under the GRM, and person estimates derived under the better‐fitting bifactor model, with respect to long‐term outcomes.

#### 3.3.1 Psychopathology

**Figure 3:**
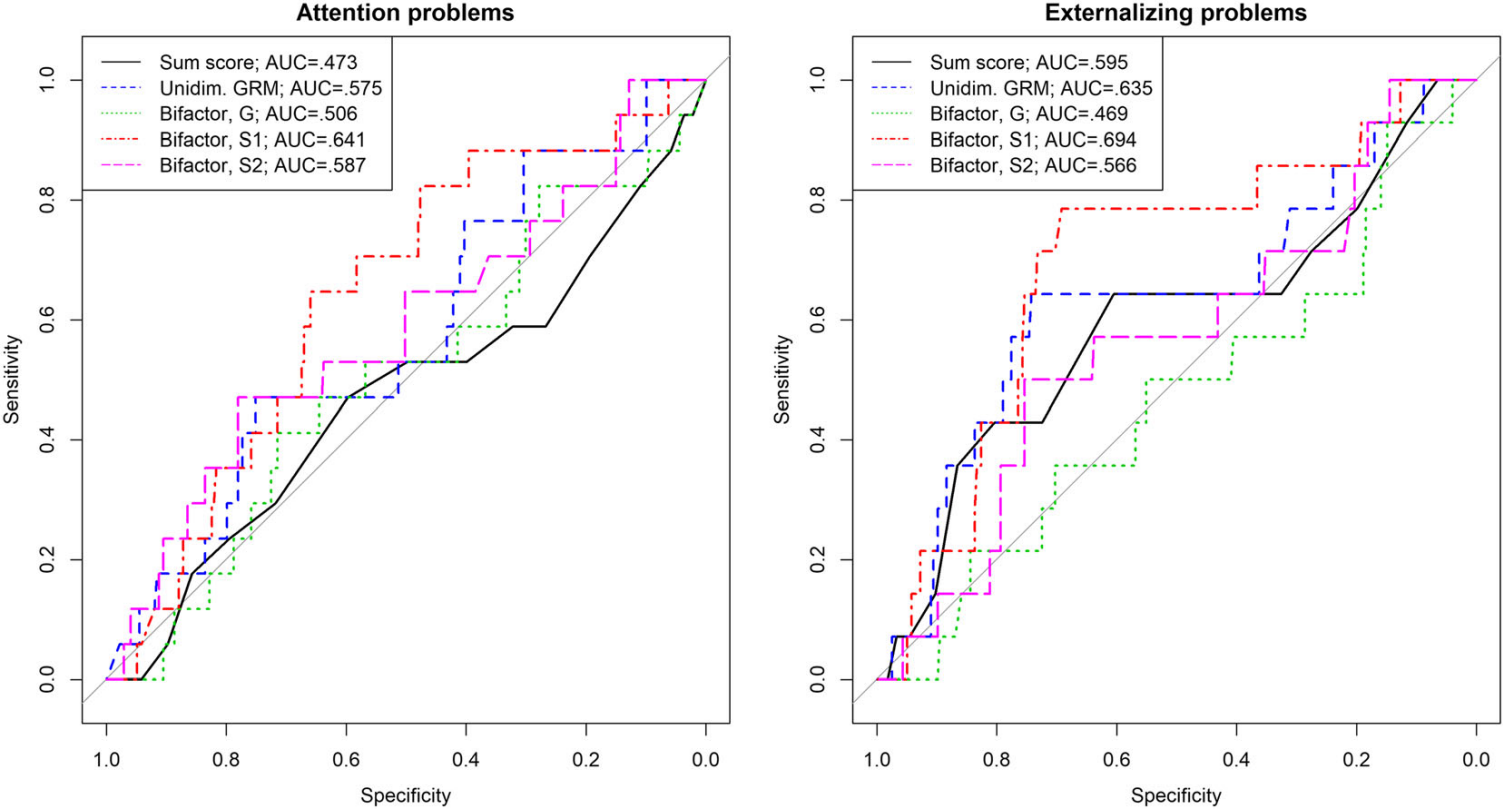
Accuracy of predicting attention problems (left panel) and externalizing problems (right panel) in young adults at T5, using attention problems severity estimates at T1. AUC, area under the curve; G, general factor; GRM, graded response model; S1/S2, specific/group factors

The AUC values in Table 6 indicate the proportion of individuals who were correctly classified as experiencing different problems in T5 (adulthood) based on the estimates of AP severity at T1 (age 11), for the three models considered. For adulthood AP (Table 6 and left panel of Figure 3), sum scores and unidimensional GRM estimates showed the lowest predictive accuracy: 47.3% and 42.2% of the individuals with clinical levels of AP at T5 were correctly classified as experiencing these problems. On the other hand, childhood AP estimates derived from the bifactor model had higher predictive accuracy, with the highest value for *S1* scores (typical ADHD symptoms). Accuracy rates for *S2* (symptoms of SCT) and *S1* scores were similar, and both estimates had higher accuracy rates than *G* (general AP) scores. For internalizing problems, we found that predictive accuracy on the basis of *S1* and *S2* was higher than those on the basis of other scores (difference in AUCs was 5.3 percentiles for *S1* and 8.6 percentiles for *S2* relative to sum scores). For externalizing problems (Table 6 and right panel of Figure 3), the results showed that scores on *S1* had the highest accuracy (67.9% correct classifications) compared with the other types of person scores.

**Table 6:** Area under the curve values indicating the predictive accuracy of each type of attention problems score estimate. Shaded cells indicate the best predictor for each outcome, in terms of the AUC

Outcome	Unidimensional		Bifactor		
	Sum scores	GRM	G	S1	S2
Psychopathology					
Attention problems (clinical)	.473	.422	.520	.627	.585
Internalizing problems (clinical)	.509	.527	.524	.562	.595
Externalizing problems (clinical)	.595	.631	.509	.679	.595
Education					
Low/low–middle	.690	.716	.545	.694	.570
Work/financial/independence					
Living with parents	.572	.574	.575	.547	.531
No paid job	.493	.523	.574	.575	.545
Low/low–middle income	.556	.590	.527	.637	.533
Social aid	.662	.641	.680	.568	.530
Relationships					
Never been in a relationship	.538	.555	.531	.596	.552

Abbreviations*:* G, general factor; GRM, graded response model; S1, first subfactor; S2, second subfactor.

#### 3.3.2 Education

For individuals with AP, educational achievement is often problematic (Fried et al., 2016). According to our data, sum scores and GRM scores performed similarly well as the scores on *S1* in terms of predictive accuracy for low, low‐to‐middle, and vocational education. The scores on *G* and *S2* had low predictive accuracy compared with the other estimates.

#### 3.3.3 Work/financial/independence

**Figure 4:**
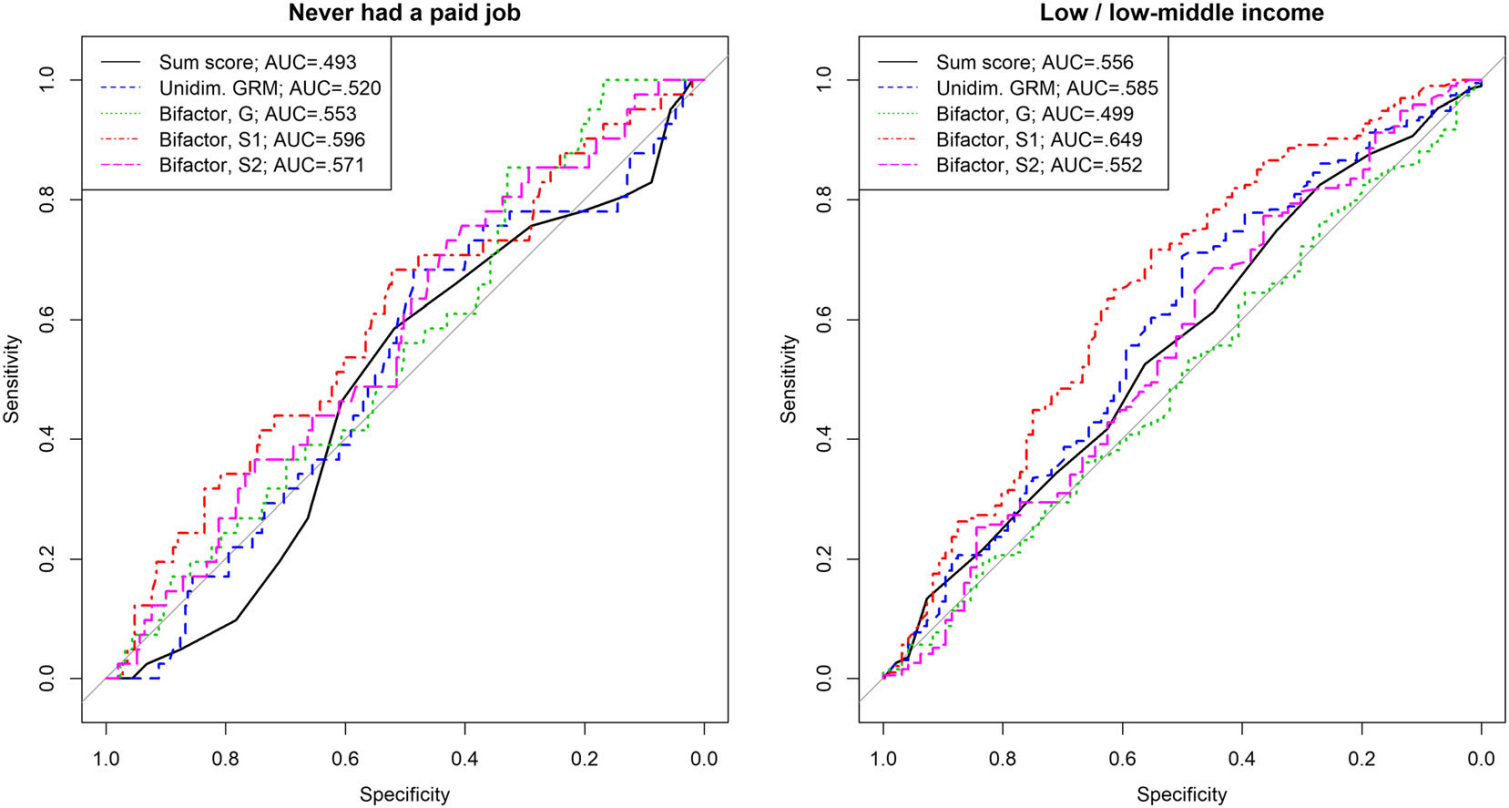
Accuracy of predicting unemployment (left panel) and low/low–middle income (right panel) in young adults at T5, using attention problems severity estimates at T1. AUC, area under the curve; G, general factor; GRM, graded response model; S1/S2, specific/group factors

Individuals with AP often encounter difficulties in finding and keeping a job and thus achieving financial independence (Brook, Brook, Zhang, Seltzer, & Finch, 2013). For the young adults who live with their parents, Table 6 shows that all person scoring strategies considered here performed similarly in terms of predictive accuracy. Overall, the accuracy of these estimates was around 55%. When predicting unemployment (Table 6 and left panel of Figure 4), there was an important increase in predictive accuracy when using score estimates from the bifactor model compared with sum scores or unidimensional GRM scores. Sum scores, GRM scores, and *G* scores performed similarly with regard to the accuracy of predicting individuals who benefit from several types of financial support from the government, whereas *S1* and *S2* underperformed in this case. Concerning the prediction of low and low‐to‐middle income (Table 6 and right panel of Figure 4), *S1* had higher accuracy compared with the other types of person scoring. Thus, the results concerning the accuracy of predicting financial status/independence based on individuals' AP severity at T1 are somewhat mixed. For some of the outcomes in this category (living with parents and social security benefits), the different models performed similarly well. For some outcomes (never had a paid job and low/low‐middle income), there was a clear advantage in using scores derived from the bifactor model.

#### 3.3.4 Relationships

For predicting individuals' ability to establish and maintain romantic relationships, results were similar for the different person scoring strategies. The predictive accuracy of these methods varied between 53% and 60% (see Table 6).

The results for predicting later‐life outcomes showed that, when comparing IRT‐derived AP scores to traditional sum scores with respect to their accuracy of classifying individuals as experiencing clinical levels of long‐term difficulties, the former tend to outperform the latter, thus supporting our hypothesis.

## 4 DISCUSSION

In this study, we investigated whether the unidimensional assumption underlying the use of sum scores to assess symptom severity holds for the Attention Problems Syndrome Scale of the CBCL/6–18 and, provided that the assumption would not hold, whether violations influence predictions of later‐life outcomes. We also investigated whether there are symptoms that functioned poorly in the scale. We used the CBCL/6–18 battery, which is an often used instrument in various high‐stake contexts. For example, the CBCL/6–18 battery is used in pediatricians' offices, schools, mental health facilities, private practices, hospitals, child and family services, public health agencies, and for research (Gregory, 2014). The Attention Problems Syndrome Scale is used to identify patients with high levels of AP (and, potentially, ADHD) who experience later‐life problems. The central question in the study was whether a more refined scoring scheme could improve the prediction of later‐life outcomes, and we hypothesized that it would.

Our psychometric analyses showed that two distinct factors underlie the 10‐item Attention Problems Scale, one tapping into the typical ADHD symptoms of inattention and hyperactivity/impulsivity and the second into behavior that we may qualify as SCT (Hartman et al., 2004; Lee et al., 2016; Becker et al., 2017; Garner et al., 2017). The distinct nature of the SCT factor was further supported by the low correlation with the factor comprising typical ADHD symptoms.

Moreover, we found that the 10 symptoms were not equally difficult and informative: Some symptoms were less common (e.g., “Stares blankly”) than others (e.g., “Fails to finish things he/she starts”), and some had higher measurement precision in the upper range of the severity continuum (e.g., “Poor school work”) than others (e.g., “Can't concentrate, can't pay attention for long”). The confirmatory analyses showed that a bifactor model with two group factors fits the data best. The symptom “Acts too young for his/her age” was found to be too general and indicative of a general developmental problem other than ADHD or SCT per se.

Knowing that multidimensionality and poorly functioning symptoms were present, we compared the traditional sum scores to scores derived from IRT models with respect to predictive accuracy. Notably, nearly all the scoring methods utilized here had AUC values lower than 0.7. Although these values indicate relatively poor predictive accuracy for the outcome measures considered here, they are quite remarkable given the long period between predictor and outcomes (more than 10 years). Considering the time span, the scores on the CBCL AP scale are good predictors for later‐life difficulties experienced by individuals with AP. For some of the outcomes (i.e., adulthood AP, internalizing problems, externalizing problems, unemployment, lower income, and inability to establish romantic relationships), we found that the scores either on the general factor or on the factor comprising typical ADHD symptoms predicted at least some of the individual outcomes with higher accuracy compared with sum scores. These findings support our hypothesis at least in part, and they are in favor of using a more appropriate person scoring strategy for these data.

The separation of the ADHD and SCT symptoms in the bifactor model in our study fits into the larger body of literature on modeling ADHD symptoms via the bifactor model (e.g., Gibbins, Toplak, Flora, Weiss, & Tannock, 2012; Gomez, 2014; Gomez, Vance, & Gomez, 2013) and into the literature examining whether SCT is a symptom of ADHD or a distinct psychopathology domain (see, e.g., Garner et al., 2017). Our findings regarding the SCT factor are in line with previous findings, in that the CBCL symptoms forming this factor had low IRT discrimination values for the general factor of AP. Moreover, when controlling for the general AP factor, the SCT scores showed higher predictive accuracy of several functional outcomes in comparison with the general AP factor. In other words, SCT scores predicted psychopathology, poor educational achievement, low‐income levels, and relationship difficulties above and beyond what was predicted by the general AP factor. Still, when controlling for general AP, the ADHD‐specific symptoms outperformed SCT with respect to predictive accuracy for most functional outcomes. Thus, further research is needed to clarify the added value of the SCT scores in predicting functional outcomes.

One of the great merits of the TRAILS study is that it provides repeated measurements more than 10 years apart. This enabled us to showcase the advantages of using a more refined scoring method for childhood AP, on predicting behavior. Our analyses showed that using a bifactor model rather than traditional sum scores to estimate AP severity in children allowed us to make more accurate predictions of several important functional criteria. The limitations of this study are inherited from the original TRAILS study and include the following (Oldehinkel et al., 2015, p. 76j): attrition at follow‐ups, low power for rare disorders and small interaction effects, and relatively small number of in‐depth assessments. Other studies found that attrition was associated with being male, low socio‐economic status, peer problems, substance use, and externalizing problems (Nederhof et al., 2012). Specific to our study, we mention the small sample sizes for the outcome variables used in predictions.

We encourage researchers to use IRT models for scale development and data analysis more often. Results in this paper showed that information can be gained over and above that provided by simple sum scores. In other words, IRT allows for a more fine‐grained picture of the construct of interest (AP in this paper). This has potential important implications for both research and practice. Our findings are in line with, and builds upon the study of, Dumenci and Achenbach (2008, p. 61), who also concluded that “resorting to summing items (i.e., CTT‐sum) may seem like a simple solution, but it invites measurement inaccuracies, especially in both tails of the distributions.” As with any statistical models, there are several shortcomings of applying IRT in the clinical field, among which we mention the relatively large sample sizes needed for optimal parameter estimation and the possibly restrictive assumptions imposed by some models on the data.

Future research can further pursue this kind of analyses for other measures of psychopathology, in order to improve measurement. To ease some of the burden of estimating IRT models, currently, there are several user‐friendly software programs for practitioners who are interested in applying IRT‐scoring procedures. Examples of such software are flexMIRT, IRTPRO, BILOG‐MG, MULTILOG, or PARSCALE, among others (e.g., various packages in the R language). Also, for detailed descriptions of IRT models, we recommend the works of Embretson and Reise (2000), Reckase (2009), or Reise and Revicki (2014). Improved measurement of psychopathology and proper scoring techniques ensure that actual decisions that are being made based on scale scores are as accurate as possible.

### DECLARATION OF INTEREST STATEMENT

The authors have no conflicts of interests to declare.

## Supporting information

Data S1. Supporting Information
