# Supplementary material for: Practical consequences of model misfit when using rating scales to assess the severity of attention problems in children
Source: Int J Methods Psychiatr Res. 2019 Jul 1;28(4):e1795. doi: 10.1002/mpr.1795 (PMC7649959; doi:10.1002/mpr.1795)
Supplement: Supplementary file 1 — Data S1. Supporting Information [file MPR-28-e1795-s001.docx]

**Online supplemental material**

Practical Consequences of Model Misfit When Using Rating Scales to Assess the Severity Of Attention Problems in Children

Table A1

*Gender-Specific Cutoff Values for Severity of Psychopathology in Young Adults*

|  | Normal | |  | Subclinical | |  | Clinical | |
| --- | --- | --- | --- | --- | --- | --- | --- | --- |
| Psychopathology | Females | Males |  | Females | Males |  | Females | Males |
| Attention problems | ≤ 13 | ≤ 14 |  | 14 – 15 | 15 – 18 |  | ≥ 16 | ≥ 19 |
| Internalizing problems | ≤ 19 | ≤ 17 |  | 20 – 24 | 18 – 23 |  | ≥ 25 | ≥ 24 |
| Externalizing problems | ≤ 16 | ≤ 18 |  | 17 – 21 | 19 – 22 |  | ≥ 22 | ≥ 23 |

**Description of IRT Models Fitted**

These are the models that were fitted to the CBCL’s Attention Problems scale at T1:

1. *The unidimensional graded response model* (GRM; Samejima, 1969). This model can be used when symptoms are measured on an ordered Likert-type scale with three or more response categories, and when one dominant factor is assumed to underlie the responses. The parameters estimated with this model for the CBCL data were: (a) A single attention problems severity score for each individual. In the population, these estimates are assumed to follow a standard normal distribution, therefore the scores can be interpreted as *z*-scores; (b) A single discrimination parameter for each symptom, indicating how well a symptom distinguishes between individuals with low and high levels of attention problems, and thus how informative the symptom is. Discrimination parameters typically range between 0.5 and 2.5 for personality items (e.g., Meijer & Tendeiro, 2012, pp. 761-762), with larger values indicating higher discrimination power and, consequently, higher measurement precision; (c) A set of 2 threshold parameters (corresponding to the three response options of the CBCL items), each of them indicating the symptom severity needed for that category or a higher category to be endorsed. Thresholds are anchored on the same scale as the severity scores and they typically range between -1.5 and 2.0 for personality items (e.g., Meijer & Tendeiro, 2012, pp. 761-762).
2. *The multidimensional GRM* (Muraki & Carlson, 1995; Reckase, 2009). This model is a generalization of the unidimensional GRM for the case when more than one factor underlies the responses. The parameters estimated with this model are: (a) A factor score for each individual on each factor, which can be interpreted as *z*-scores; (b) A set of discrimination parameters for each symptom, one parameter for each factor; (c) A single set of 2 location parameters for each symptom. In this model, the factors are allowed to correlate.
3. *The full-information bifactor model* (Gibbons & Hedeker, 1992; Gibbons et al., 2007). This model consists of a general factor (attention problems) and two or more uncorrelated specific (or group) factors. According to this model, each symptom is assumed to have a non-zero loading/discrimination parameter on the general factor and at most one discrimination parameter on one of the group factors. The group factors represent common sources of variance in the data, controlling for the general factor (Reise, Bonifay, & Haviland, 2013). The parameters obtained from fitting this model to these data were: (a) A set of factor scores for each individual in the dataset, one value for each of the general and the group factors; (b) One or two discrimination parameters for each symptom - one parameter corresponding to the general factor and (possibly) one parameter corresponding to a group factor; (c) A single set of 2 location parameters for each symptom.

**Description of the Fit Indices Used**

- *The M2^*^ limited information statistic*. For this statistic, a non-significant result indicates lack of evidence of model misfit (Cai & Hansen, 2013);
- *The sample bivariate root mean square error of approximation (RMSEA)*. Values $\leq$ 0.05 indicate good fit (Maydeu-Olivares & Joe, 2014);
- *The standardized root mean square residual (SRMSR)*. Values $\leq$ 0.05 indicate good fit (e.g., Hu & Bentler, 1999; Liu & Maydeu-Olivares, 2014);
- *The comparative fit index (CFI)*. Values $\geq$ 0.95 indicate good fit (e.g., Hu & Bentler, 1999);
- *The Tucker-Lewis index (TLI)*. Values $\geq$ 0.90 indicate good fit (e.g., Hu & Bentler, 1999);
- *The Akaike Information Criterion* (AIC; Anderson, Burnham, & Thompson, 2000) and the *Bayesian Information Criterion* (BIC; Raftery, 1995) for model comparisons. For these fit indices, lower values indicate better fit.
